# Nitrate and Nitrite in Health and Disease

**DOI:** 10.14336/AD.2017.1207

**Published:** 2018-10-01

**Authors:** Linsha Ma, Liang Hu, Xiaoyu Feng, Songlin Wang

**Affiliations:** Salivary Gland Disease Center and Beijing Key Laboratory of Tooth Regeneration and Function Reconstruction, Capital Medical University, Beijing 100069, China; Salivary Gland Disease Center and Beijing Key Laboratory of Tooth Regeneration and Function Reconstruction, Capital Medical University, Beijing 100069, China; Salivary Gland Disease Center and Beijing Key Laboratory of Tooth Regeneration and Function Reconstruction, Capital Medical University, Beijing 100069, China; Salivary Gland Disease Center and Beijing Key Laboratory of Tooth Regeneration and Function Reconstruction, Capital Medical University, Beijing 100069, China

**Keywords:** dietary nitrate, NO_3_^-^-NO_2_^-^-NO pathway, circulation, sialin

## Abstract

The source of dietary nitrate (NO_3_) is mainly green, leafy vegetables, partially absorbed into blood through intestinal mucosa. The recycled nitrate is reabsorbed and concentrated by the salivary glands and then secreted into saliva. In 2012, sialin was first discovered as the mammalian membrane nitrate transporter in salivary glands and plays a key role in circulation of inorganic nitrate, providing a scientific basis for further investigation into the circulation and functions of nitrate. Dietary nitrate can be converted to nitrite (NO_2_) by oral commensal bacteria under the tongue or in the stomach, following which nitrite is converted to nitric oxide (NO) through non-enzymatic synthesis. Previously, nitrate and nitrite were thought to be carcinogenic due to the potential formation of nitrogen compounds, whereas the beneficial functions of NO_3_^-^-NO_2_^-^-NO pathway were ignored. Under conditions of hypoxia and ischemia, the production of endogenous NO from L-arginine is inhibited, while the activity of exogenous NO_3_^-^-NO_2_^-^-NO is enhanced. Recently, a greater amount of evidence has shown that nitrate and nitrite serve as a reservoir and perform positive biological NO-like functions. Therefore, exogenous dietary nitrate plays an important role in various physiological activities as an effective supplement of nitrite and NO in human body. Here we generally review the source, circulation and bio-functions of dietary nitrate.

Nitrate (NO_3_) and nitrite (NO_2_) widely exist in water, soil, air, and plants [1]. The main source of absorbed nitrate in the body is food, with green vegetables contributing the major portion. Although nitrates are stable, dietary nitrate is converted to nitrite through a non-enzymatic process and nitric oxide (NO) by symbiotic bacteria in the oral cavity and stomach, therefore performing physiological NO functions. NO, the metabolic product of dietary nitrate, plays an important role in protecting the cardiovascular system and gastric mucosa, and in metabolic diseases [2, 3]. Endogenous NO is derived from the arginine pathway and is regulated by nitric oxide synthase (NOS) and its redox state. However, under conditions of hypoxia and ischemia, the activity of NOS is down-regulated resulting in a decreased production of endogenous NO. The skeletal muscle cells of rats were found to be capable of nitrate intake from peripheral blood, following which nitrate was deoxidized to NO by xanthine oxidation-reductase pathway, thereby increasing blood flow rate and enhancing metabolism [4]. Dietary nitrate served as an effective donor of NO, and the possible functions of NO from dietary nitrate are being widely studied.

Nitrate was thought to be harmful due to the potential production of carcinogenic nitrosamines under certain conditions such as an acidic stomach. Nitrosamines were reported to be related to esophageal cancer, gastric cancer, colon cancer, and other tumors [5, 6]. Thus, the World Health Organization (WHO) recommended the upper limit of concentration of daily nitrate and nitrite uptake to be 3.7 mg/kg and 0.06-0.07 mg/kg, respectively [4]. However, recent epidemiological investigations of nitrates and tumors have shown that no clear evidence has verified that dietary nitrate could increase the occurrence of tumors [7]. In 2012, sialin was first reported as a nitrate cell membrane transporter which played an important role in the circulation of dietary nitrate. Nitrate is actively transported by sialin in salivary glands, concentrated in saliva, and then secreted into the oral cavity, after which it reenters body circulation through the stomach and intestine [8]. As dietary nitrate is converted to NO by oral and stomach bacteria through non-enzymatic synthesis, nitrate could be considered indispensable in physiological activities.

## Source of nitrate and nitrite

Systemic circulating nitrate is mainly obtained from two sources, diet and oxidation of endogenous NO, which correspond to exogenous and endogenous nitrates, respectively [9]. Exogenous sources of nitrate for human intake are primarily foods which account for approximately 60%-80% of the total nitrate intake [10]. In keeping with recent reports, vegetables, especially green leafy vegetables, such as spinach and beetroot contain an abundance of nitrate [11], which contributes nearly 80%-90% of the total dietary nitrate [12]. Other sources of nitrate are drinking water (15%-20%) and other foods, including animal-based products (10%-15%) [13].

With respect to nitrite, approximately 80%-85% [9, 14] of total systemic nitrite is obtained through endogenous conversion from nitrate [15]. Nearly 93% nitrite is converted from nitrate [16]. An individual consumes about 1.2-3.0 mg nitrite every day [17]. The other sources of nitrite are oxidation of endogenous NO and exogenous nutritional sources (cured meats comprise 4.8% and vegetables account for 2.2%) [10]. Exogenous nitrite is almost completely absorbed in the duodenum and jejunum [18]. Most systemic circulating nitrite is converted to NO and serves as a relatively stable reservoir of NO.

## Distribution and conversion of nitrate and nitrite

Nitrate and nitrite exist widely in the human body, while the distribution is quite different. Volunteers receiving water labeled nitrogen 13 (^13^N0_3_^-^) were found that nitrate did not rapidly absorbed into blood from the stomach but rather stably existed in the intestine. While after intravenous administration of ^13^NO_3_^-^, the distribution of nitrate was active in heart, reaching peak concentration of about 3% percent of total nitrate at 2 minutes, then fell rapidly in the next 2 minutes [19, 20].

Systemic nitrate and nitrite was circulating among blood, saliva and tissues, after a rich nitrate diet, the nitrate was absorbed and the plasma level peak up in 15-30 minutes with a half-life of about 5-8 hours [3, 21, 22]. As the concentration of nitrate was about 10 times of that in plasma, saliva contained large amount of total nitrate [23]. The active ingestion ability of nitrate in different organs differs greatly, possibly depending on the expression of nitrate transporter protein-sialin [8, 24].

Nitrite in blood soon converts to nitrate with half-life about 110s, while nitrite in plasma is relatively stable with half-life about 20-30 mins [4, 25-28]. Normal plasma levels of nitrite are 50-100 nM and increase 4-5 times after a nitrate-rich meal, in which process numerous proteins and enzymes in blood and tissues catalyze the reduction of nitrate to nitrite [2, 29]. The conversion of nitrate to nitrite was an enzymatic process, while the conversion of nitrite to NO was a non-enzymatic process.

## Circulation of nitrate and nitrite

**Figure 1.**
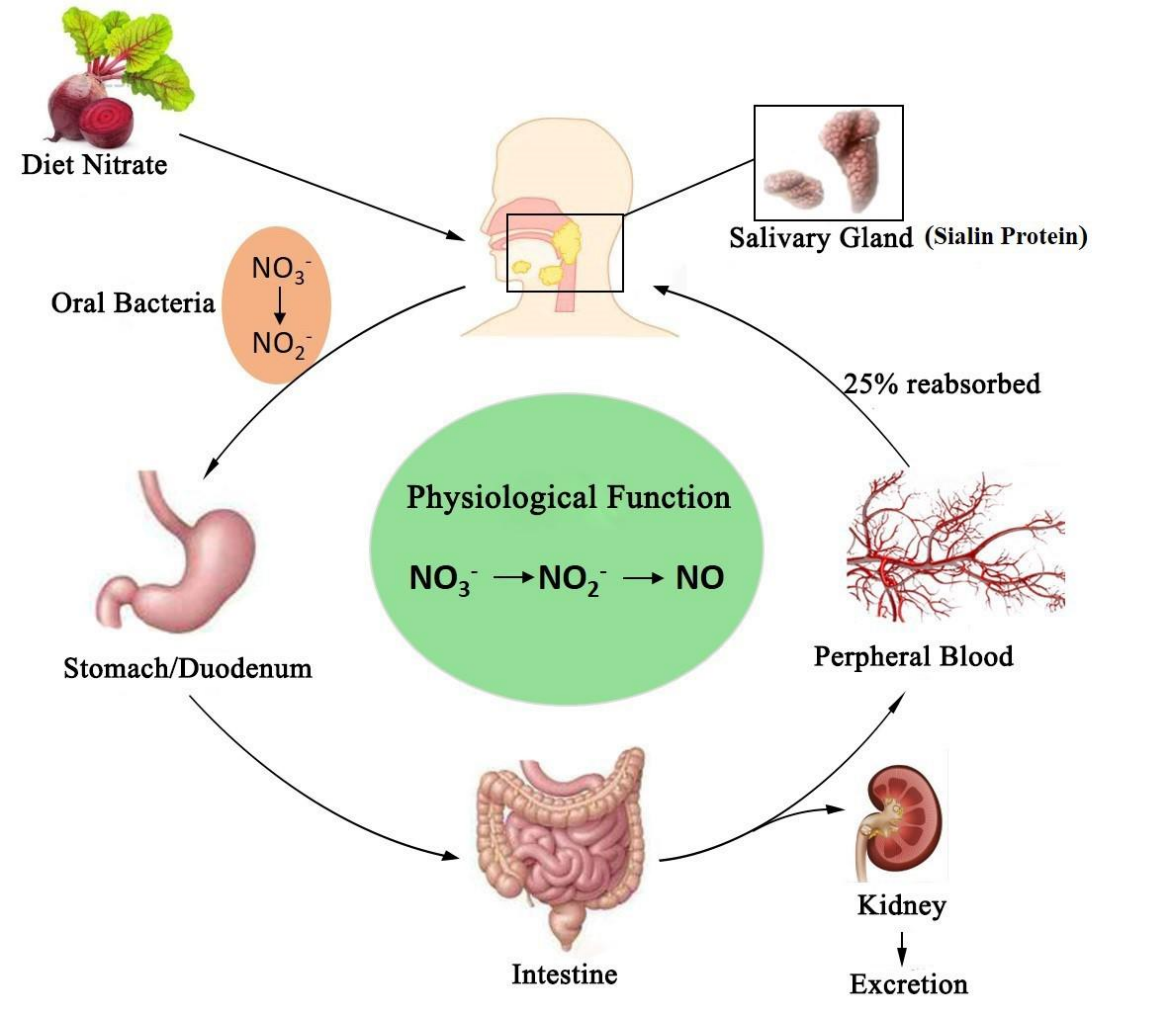
Circulation of nitrate in the body The recycling of dietary nitrate is mainly in salivary glands, where sialin plays a key role in active transport and concentration of nitrate. Part of the nitrate is converted to nitrite by oral bacteria and subsequently absorbed in the stomach and intestine. Nearly 25% of circulating nitrate is reabsorbed by the salivary glands, whereas the majority is excreted by the kidneys. Nitrate performs physiological functions through the exogenous NO_3_^-^-NO_2_^-^-NO pathway. NO, nitric oxide; NO_2_^-^, nitrite; NO_3_^-^, nitrate.

The salivary glands and oral bacteria play an essential role in the circulation and conversion process of exogenous NO_3_^-^-NO_2_^-^-NO pathway. Dietary nitrate is absorbed almost entirely owing to its bioavailability in the stomach and the small intestine, and about 75% is excreted in urine, while the remaining amount is reabsorbed in the kidney, by biliary and in salivary glands [3, 30, 31]. Under normal conditions, up to 25% of recycled nitrate can be found in salivary glands, where the nitrate concentration reached 10 times that of the plasma [32]. In 2012, based on the organ model of salivary glands, sialin was discovered as the nitrate transporter in mammalian cell membranes, which provided the scientific foundation for the study of the biological effect and metabolism of nitrates in the body [8, 24, 33] Approximately 5%-7% of dietary nitrate is converted to nitrite in the oral cavity by commensal facultative anaerobic bacteria located in the deep crypts of the posterior part of the tongue [34, 35]. Thereafter, most nitrite is converted to nitric oxide in the stomach and absorbed systematically (Fig. 1).

## Function of nitrate and nitrite

Under conditions of hypoxia and ischemia, the production of endogenous NO from L-arginine is inhibited. On the contrary, the activity of exogenous NO_3_^-^-NO_2_^-^-NO is enhanced. Thus, dietary nitrate and nitrite serve as effective donors of NO under conditions of hypoxia and ischemia [34]. Nitrate and nitrite are used as food additives in processed food where they act as preservatives by inhibiting the growth of microorganisms, notably *Clostridium botulinum*. Besides the direct anti-microbial effect of nitrite, the physiological effects of nitrogen species including inorganic nitrate and nitrite have been reported recently.

When dietary nitrate is not available, the excretion of total nitrate calculated in health volunteers was much larger than the amount of intake, indicating that nitrate and nitrite could be formed by endogenous synthesis. The endogenous production of nitrate was mainly in intestine mucosa tissues [36]. Nitrate performs physiological functions in various systemic activities, including blood pressure reduction, platelet aggregation inhibition, and vessel protective effect - functions similar to those of NO [3, 37]. Nitrate prevents ischemic heart disease by increasing epicardial blood flow through vasodilation, decreasing vascular resistance, blunting coronary steal, and reducing preload [38]. Dietary nitrate (10 mmol/L soldium nitrate in drinking water) can partly improve age-related hypertension and metabolic activities in mice through a decrease of endogenous NO generation via inhibition of NADPH oxidase and modulation of angiotensin (ANG) II receptor expression [39]. Furthermore, inorganic nitrates suppress (15 mmol/L KNO_3_) acute and chronic inflammation by raising the neutrophil count, which may reduce the occurrence of atheromatous plaque [40]. Moreover, a long-term dietary nitrate and nitrite deficiency experiment showed that mice would suffer from metabolic syndrome, endothelial dysfunction, and cardiovascular death after 22 months of a low-nitrite/nitrate diet [41]. Inorganic nitrate performs functions of decreasing blood pressure and improving myocardial ischemia by enhancing epithelial cell activity and diastole blood vessels, and reducing platelet aggregation [42].

Nitrates secreted from saliva protect against gastric ulcers by promoting gastric NO expression and stimulating concomitant mucus formation [43]. Stress-induced gastric damage was reported with a water immersion-restraint stress (WIRS) assay in a rat model. Results showed that stress promotes salivary nitrate secretion and nitrite formation in health volunteers, and that exogenous nitrate administration (5 mmol/L NaNO_3_) recovered gastric mucosal blood flow and introgastric NO level, thereby rescuing the WIRS-induced gastric damage [44]. The concentration of bioactive NO in the stomach increased 50-fold after ingestion of dietary nitrate [45]. Meanwhile, the non-enzymatic production of NO from dietary nitrate (0.1 or 1 mmol/kg NaNO_3_) could effectively alleviate diclofenac-induced stomach mucosa injury and improve the thickness of slime layer in the stomach [43].

NO could regulate metabolic disorder-induced cardiovascular diseases and other metabolic disease. Moreover, the synthesis of NO was reduced in obese mice [46]. Metabolic disorder-induced high blood pressure, insulin resistance, and carbohydrate tolerance were found in ENOS (endothelial nitric oxide synthase)-knockout mice [47]. Dietary nitrate effectively supplements NO by the activated exogenous NO_3_^-^-NO_2_^-^-NO pathway under conditions of hypoxia. With a continuous 3-day supplement of 0.1 mmol/kg sodium nitrate pre-exercise, the oxygen consumed was reduced to an average of 5% [48]. Besides, nitrate enhanced the exercise tolerance of health volunteers by NO-cGMP-PPAR pathway and increased the metabolism of fatty acid in skeletal muscle cells [49]. Exogenous nitrate (0.7 mmol/L NaNO_3_) could active the cGMP pathway in mice and promote the conversion of white adipose to brown adipose, therefore enhancing fat metabolism and decreasing body weight [50]. As metabolic diseases were mostly accompanied with NO synthetic disorder, the supplement of exogenous NO from dietary nitrate could partially alleviate metabolic diseases. Therefore, exogenous dietary nitrate is an essential element in the human body and plays an important role in various physiological activities as an effective supplement of nitrite and NO.

## Safety of nitrate and nitrite

Previously nitrate and nitrite were considered as precursors of N-nitroso compounds that were classified as human carcinogens. Nitrate was converted to nitrite and then gets into the stomach, where the condition of low pH promotes the conversion of nitrite to reactive nitrous acid [51]. Besides under conditions of inflammation and bacteria (Helicobacter Pylori), the formation of nitrate related nitrosamine was enhanced. Patients with achlorhydria and bacterial overgrowth were at high risk of developing gastric cancer possibly because of the formation of nitrosamine [52, 53]. However, this process would be weakened by polyphenols and other antioxidants such as vitamin C. With enough amounts of antioxidants as vitamin C, the nitrosylation of secondary amine through nitrite was inhibited [54, 55].

The International Agency for Research on Cancer (IARC) has concluded that there was no substantial evidence implicating nitrates as animal carcinogens in 2010 [56]. Moreover, in recent epidemiological investigations, dietary nitrate showed no association with gastric cancer or esophageal cancer in humans [7, 57]. Some research even showed that nitrate could decrease the occurrence of gastric cancer [11, 58], possibly because the main source of dietary nitrate are vegetables, which contain a large amount of fiber, vitamin C, and other reductants. An investigation in Korea, where the intake of dietary nitrates (390-742 mg/day) is considerably higher than that of European countries (52-156 mg/day) and China (422.8 mg/day), showed that no correlation was found between high intake of nitrate and cancer [59]. Besides, the safety of high dietary nitrate (91 g/L potassium nitrate) supply was identified in a miniature pig model. Liver and kidney tissues were checked after high-dose nitrate feeding for 2 years, and no observed systemic toxicity or damage was found in miniature pigs [60]. With 17 continuous weeks of 85 mg/L sodium nitrate-water supplement, increased insulin sensitivity, decreased plasma IL-10 level, and tendency of pro-long lifetime were found without body injury in these mice [61].

The association of nitrite with cancer seems conflicted [11]. The correlation between nitrite and gastric cancer is contradictory in different epidemiological surveys [57]. In 2011, carrying out a large cohort study including approximately 50000 individuals, followed up on for almost 10 years, Cross and his teammates concluded that nitrate and nitrite were not associated with esophageal or gastric cancer, whereas positive associations were found between red meat intake and esophageal squamous cell carcinoma [62]. Some epidemiological studies use processed or smoked meat as a source of exogenous nitrite ignoring complex compounds such as nitrosamines in such foods, resulting in lack of uniformity and scientific accuracy in conclusions. Therefore, association of exogenous nitrite with cancer seems less likely because large amounts of nitrite are formed endogenously. The nitrite concentration in saliva may rise as high as 72 mg/L after consumption of nitrate equivalent to 200 g of spinach [63]. Besides, people are in contact with nitrosamine in many circumstances, such as through smoke, beer, water, working environment, especially cigarettes which contain about 100-1000 times the level of nitrosamine in the daily diet.

Methemoglobinemia was found to be caused on ingestion of excessive nitrite [64], whereas ingestion of excessive nitrate did not lead to the disorder. A study in America showed that even though the mother ingested a large amount of nitrate, the baby would not get methemoglobinemia through breast feeding [65]. On the other hand, dietary nitrate, the source of which is mostly vegetables, which contain a large amount of antioxidants, effectively decreases the occurrence of methemoglobinemia [3].

## Clinical application of nitrate and nitrite

Under conditions of illness or senescence, the activity of eNOS was reduced and the production of NO was decreased, as reported [46, 66, 67], thus indicating exogenous source of NO supplement might have a potential therapeutic treatment for patients undergoing illness or senescence. Nitrate was reported could lower blood pressure in health volunteers [68] and established in several experiments in human trials [69]. The aortic pulse wave velocity was improved, and the platelet-monocyte aggregates reduced in nitrate supplemental patients which resulting in decreased blood pressure [70]. Whilst with positive clinical trials, negative results were reported showing no significant effect was found in lowing blood pressure with nitrate supplement [71, 72]. The reason of these complex results was uncertain and more clinical trials were needed for further research.

Although many positive effects were found in animal models, the clinical usage of nitrate was quite limited. In recent years, nitrate-rich fruit and vegetable drinks especially beetroot drinks, Biotta veggie drink (Biotta®, Switzerland) [73], and BEET-IT (James White Drinks, Ipswich, UK) [74-76] have become popular. With increasing awareness on nitrates, nitrates and nitrate-related products are being accepted.

## Conclusion

As part of their daily diet, people ingest inorganic nitrate mostly through green, leafy vegetables. Apart from their negatives, dietary nitrate and nitrite have been reported as exogenous donors of biological NO, playing an important role in physiological activity. Furthermore, dietary nitrate supplements seem to have potential protective effect for body balance, improvement of disorders (stroke, myocardial infarction, systemic and pulmonary hypertension, etc.), and in alleviation of gastric ulcers. Normal dietary nitrate and nitrite showed no harm to human health and no confirmed evidence stated the explicit association of dietary nitrate and cancer. Most existing research on nitrite and tumors ignored the complicated compounds in target foods, resulting in contradictory conclusions among researchers. Considering the various protective effects, other than the formal harmful suspects, dietary nitrate and nitrite play an important role in physiological functions through the provision of non-enzymatic NO. With a new understanding of nitrates and nitrites, their biological functions and applications need further investigation in the future.
